# UV filters and high refractive index materials based on carboxymethyl cellulose sodium and CuO@ZnO core/shell nanoparticles

**DOI:** 10.1038/s41598-023-48345-5

**Published:** 2023-11-30

**Authors:** Rania Badry, Mahmoud M. El-Nahass, Nadra Nada, Hanan Elhaes, Medhat A. Ibrahim

**Affiliations:** 1 Physics Department, Faculty of Women for Arts, Science and Education, Ain Shams University, Cairo, 11757 Egypt; 2 Physics Department, Faculty of Education, Ain Shams University, Roxy, Cairo, Egypt; 3 Spectroscopy Department, National Research Centre, 33 El-Bohouth St., Dokki, Giza, 12622 Egypt; 4 Molecular Modeling and Spectroscopy Laboratory, Centre of Excellence for Advanced Science, National Research Centre, 33 El-Bohouth St., Dokki, Giza, 12622 Egypt

**Keywords:** Materials science, Physics

## Abstract

Nanoparticles have substantially contributed to the field of skincare products with ultraviolet (UV) filters to preserve human skin from sun damage. Thus, the current study aims to develop new polymer nanocomposites for the efficient block of UV light that results from the stratospheric ozone layer loss. Co-precipitation method was used to successfully synthesis CuO@ZnO core/shell NPs with a well-crystalline monoclinic CuO core and wurzite ZnO shell. Using the casting method, core/shell NPs were successfully introduced to carboxymethyl cellulose sodium (CMC). The CMC nanocomposites displayed considerably broader optical response extending from near-ultraviolet to visible light, which was likely due to heterojunction between the p-CuO core and n-ZnO shell and defects originating from the synthetic process. The transmittance of pure CMC in the UV, visible, and near IR regions is significantly reduced with the addition of 2 and 4 wt% of CuO@ZnO core/shell NPs to CMC. 99% of UV light is absorbed when 4 wt% of CuO@ZnO core/shell NPs are added. The addition of different concentrations of CMC nanocomposite to one of the sunblock in Egyptian market were studied and showing the highest Sun Protection Factor of 22. Moreover, optical dispersion parameters and refractive index were improved strongly with core/shell NPs addition.

## Introduction

There is a high demand for biodegradable and renewable UV protection films to satisfy the increasing sustainable demands of the environment. Nowadays, the world is witnessing a crisis in preventing the harmful effects of UV radiation due to the hole in the ozone layer^1,2^. As the hole in the stratospheric ozone layer is mainly caused by the human activities without taking environmental considerations into account; where scientists discovered that there is a group of very inert chemicals that are the main cause of the depletion of the ozone layer, when these materials reach the stratosphere, chloride, fluoride and bromide atoms are released under the effect of UV radiation, and begins the process of decomposing ozone gas^3,4^. In recent decades, the depletion of the stratospheric ozone layer has led to a gradual increase in UV radiation, which increases harmful side effects to human health, such as skin burns, premature aging, immune system damage, and skin cancer^5^. UV radiation is classified into three regions: ultraviolet-A (UVA: 320–400 nm), ultraviolet-B (UVB: 280–320 nm), and ultraviolet-C (UVC: 200–280 nm). UVA accounts for nearly 90–99% of UVR that reaches the earth's surface, while UVB accounts for only 1–10%. However, UVC rays are completely filtered out by the oxygen atoms in our atmosphere. As a result, the number of skin care products that contain UV filters has increased rapidly, which helps protect human skin from sun damage^6^.

However, there are concerns about the active compounds in commercially available sunscreens. Sunscreens are made up of organic materials, inorganic materials, or a combination of the two. Organic compounds like avobenzone and octocrylene have been linked to endocrine disruption and skin irritation. Furthermore, UV rays easily decompose these organic compounds, resulting in a decrease in UV-absorbing efficiency over time^7,8^.

Because of their biocompatibility, inorganic ingredients such as titanium dioxide (TiO_2_) and zinc oxide (ZnO) are generally regarded as safer than their organic counterparts, but they also have drawbacks such as being opaque and photocatalytic^9,10^. Because of its physical stability and non-toxicity, ZnO is typically regarded as one of these semiconductors' most promising photo-catalytic material. Its high oxidant power and hydrophobicity are responsible for its strong antibacterial action^11^. ZnO-NPs have been shown to absorb some UV light and convert it to harmless infrared light^12^. Because of their high transparency in the visible region of the electromagnetic spectrum and excellent UV absorption, these metal oxide nanoparticles are commonly used in UV-protection sunscreen and coating applications^13,14^. MONP's global market is expected to grow rapidly in the coming years as its numerous benefits are recognized.

Electrons are stimulated to the conduction band (CB) of ZnO when exposed to solar radiation with energy greater than its band gap, leaving holes in the valence band (VB). Once on the ZnO surface, the photo-generated electrons and holes produce highly reactive oxygen species (ROS), like the peroxide anion (·O_2_^·−^) and the hydroxyl radical (·OH) that causes the photo-degradation of pollutants. However, because of its large band gap, ZnO is only active when exposed to UV light. Due to ZnO photocatalysts' poor solar energy conversion efficiency and the fact that solar light only contains 5–7% UV radiation, their wide-scale application is severely constrained. Additionally, the high rate of photogenerated e^−^/h^+^ pair recombination in ZnO, results in low quantum efficiency which significantly lowers photocatalytic activity. Therefore, it is critically necessary to increase the separation efficiency of ZnO’s e^−^/h^+^ pairs and to extend visible light responsiveness. It is a suitable way to demonstrate the photocatalytic activity of ZnO by adding other transition metal ions because the dopant metals provide new energy levels within the band gap that largely enhance the photocatalytic activity^15^.

Although inorganic filters have solved the opaqueness problem by reducing particle size to the nanoscale, the photocatalytic issue persists when inorganic ingredients are used in sunscreens. Under UV illumination, photocatalytic reactions produce highly reactive oxygen species (ROS), which harm healthy skin cells. To avoid potential harm to individuals as a result of sunscreen toxicity or phototoxicity, non-toxic and photostable UV- absorbing agents must be developed^16–18^.

By appropriately doping specific elements, it is possible to improve ZnO performance and increase its light absorption across the visible and ultraviolet spectrum by decreasing its bandgap. The use of non-metal elements as dopants^19,20^, the noble metal nanoparticle’s deposition (Au, Pt, Ag), or the use of other semiconductors are some of the methods that have been suggested. The fabrication of a distinct and well-defined core/shell nanoparticle by combining CuO and ZnO in such manner that either ZnO is deposited onto CuO (CuO@ZnO) or reversely by depositing CuO onto ZnO (ZnO@CuO) has shown to be a successful process that has been tested numerous times^21–25^.

Because they have more potential uses than separate components, core/shell NPs have recently attracted a lot of attention^26^. Due to the attractive p-type material's semiconducting properties and high absorption coefficient, the coating or shell creation of ZnO to CuO nanoparticles offers a strong opportunity^27,28^. CuO/ZnO heterojunctions are being viewed as effective and promising building blocks in the recent times for sensors, storage devices, water treatment and solar-based photocatalytic reduction^29^.

Because of their high contact area at the p–n junction interface and high surface area to volume ratio, which permit more electron–hole formation, the core/shell nanoparticles are anticipated to have better properties. As a result, there is a lot of interest in the development of core/shell nanoparticles, that improved charge collection efficiency, for applications in optoelectronic and nanoelectronics devices. Furthermore, p-CuO@n-ZnO core/shell heterojunction at the nanostructure interface may develop advantageous radial p-n junctions, which could improve charge collection, lower interface defect states, and ultimately lead to better performance in nanoelectronics devices. Although both homogeneous ZnO and CuO nanostructures have been extensively investigated, the exploration of the CuO/ZnO related heterogeneous nanostructures is still inadequate. Furthermore, most of the core/shell structured CuO/ZnO nanomaterials employ the ZnO^30,31^ as the core rather than the CuO^32^.

Synthetic polymers are the primary substrate of UV blocking. Because of the drawbacks of petroleum-based polymers, developing a green and sustainable generation of transparent UV filters that are self-protective and made from natural materials is critical^33–36^. UV absorber-containing cellulose-based films have been reported to have potential applications as outdoor UV-sensitive polymers, car windscreens, clean windows, contact lenses, special biological test containers, and as an alternative to synthetic polymers^37–40^.

Because of its strong inter- and intramolecular hydrogen bonds and partially crystalline structure, cellulose is difficult to melt and dissolve in water^37^. As a result, cellulose-based films continue to have limitations such as brittleness, poor mechanical behavior, and water sensitivity^41,42^. The cellulose conversion into carboxymethyl cellulose sodium (CMC) clearly improves its film-forming characteristics as a hydrophilic polysaccharide. The attached carboxymethyl group disrupts the hydrogen bonding between the cellulose chains and thus can form hydrogen bonds with water molecules^43^.

Carboxymethyl cellulose sodium is one of cellulose derivatives and a water-soluble lignocellulosic material with carboxy methyl groups attached to some of the hydroxyl groups of the cellulose backbone's glucopyranose monomers^44^. CMC has attracted scientific interest because of its polyelectrolyte properties. It is discovered to be effective for applications such as packaging material (for coatings and films), thickening agent, flocculating agent, emulsifier, medicine, water-retaining agent, chelating agent, and sizing agent due to its good film-forming property, high transparency, biodegradability, and non-toxicity^45,46^. Because of its low cost and high stability, this anionic polymer has been used in a variety of applications, including cosmetics, food, paint, oil drilling, textiles, detergents, and pharmaceuticals^46–48^. CMC has been frequently used in the synthesis of gold, silver, and platinum NPs as a supportive material or a reductant. Few researches have recently investigated the usage of CMC composites as hydrogen, humidity, and liquid petroleum gas sensors^48^. Because CMC has a high viscosity at a low concentration, it is used as a food additive to improve food stability and thickness. CMC's biodegradability and biocompatibility have attracted the attention of biomedical researchers, and it has primarily been used in the form of hydrogels in areas such as tissue engineering, drug delivery, and wound dressing^49,50^. The UV protection of lignin in paint and varnish has been reported as a more environmentally friendly alternative to synthetic UV blockers. Finally, it has been reported that lignin can be used as a UV shield to protect microorganism^34^.

This paper discussed the preparation of CuO@ZnO core/shell nanoparticles using co-precipitation method and then characterize them structurally by X-ray diffraction (XRD), high-resolution transmission electron microscopy (HR-TEM), and Fourier transform infrared spectrophotometer (FTIR) was utilized to determine the functional groups of the CuO@ZnO core/shell nanoparticles. However, the major aim of this work is to improve the optical properties of CMC by incorporating different weight percentages of CuO@ZnO core/shell NPs to improve its UV blocking properties. To the best of our knowledge, no research has been conducted on how the concentration of CuO@ZnO core/shell NPs affects the optical properties and UV blocking capacity of CMC.

## Experimental section

### Chemicals

The reagents in the following list were all used without further purification. El Nasr Pharmaceutical Chemicals Co., Cairo, Egypt provided the sodium hydroxide pellets and absolute ethanol (analytical reagent, 99.9%). While copper chloride (laboratory reagent, 99%) was acquired from Sigma-Aldrich, Germany. Carboxymethyl cellulose sodium (CMC) with Mw = 2.5 × 10^5^g/mol was purchased from K. Patel Chemo pharma PVT, India. The glassware was completely washed with distilled water (D.W.) after being properly cleaned with soap solution. Distilled water was used to prepare all solutions. In particular, the creation of the ZnO shell onto the core CuO NPs and the synthesis of the CuO@ZnO core/shell nanoparticles are two sequential stages.

### CuO preparation

CuO NPs were prepared using the same procedures reported in reference^51^. In 100 mL of (D.W.), 0.2 M copper chloride was dissolved and stirred using a magnetic stirrer. This solution was supplemented with 0.4 M sodium hydroxide (NaOH) pellets, and the combination was heated to roughly 50 °C for four hours while maintaining a pH of eight. After a while, black precipitates will begin to collect at the beaker's bottom. The particles were collected, centrifuged for 10 min at 8000 rpm, and then washed three times with D.W. and once with ethanol. Now the residue collected was carefully moved to an oven and heated to about 80 °C for drying (for 2 h) and then calcined at 500 °C for 2 h to produce CuO nanoparticles. The method was repeated in order to obtain CuO-NPs, which were required for the following step.

### CuO@ZnO core/shell preparation

The Co-precipitation method was used to create the CuO@ZnO core/shell nanoparticles. After adding 100 mL of D.W., 2.19 g of zinc acetate aqueous solution was added in one beaker until zinc acetate dissolves, the mixture was left at 50 °C on a magnetic stirrer. 0.2 g of the previously produced CuO-NPs were mixed with 40 mL of D.W. in a different beaker. The CuO solution was exposed to ultra-sonication for 30 min to achieve good dispersion of the CuO-NPs. The zinc acetate beaker was filled with CuO solution and agitated for approximately 15 min. To the previously heated solution, approximately 0.2 M of NaOH is added until the pH becomes between 10 and 11 (approximately stirred for 6 h). The considerable volume of grey precipitate is created instantly. It is centrifuged and cleaned with D.W. three to four times. The precipitate was produced, dried in a dryer for two hours at 70°°C, and then calcined for a further two hours at 500°°C.

### CMC/CuO@ZnO core/shell nanocomposite preparation

The production of virgin CMC and CMC/ CuO@ZnO nanocomposite films was carried out using the solution casting method, the same method used in our previous work to prepare CMC/CuO nanocomposite. To create a homogenous CMC solution, 0.5 g of carboxyl methyl cellulose was first immersed in 100 mL of distilled water while being continuously stirred at 40 °C for 4 h. The film solution was cast into two glass petri dishes after cooling it to room temperature and removing any air bubbles. The solution was dried for 24 h at 40 °C in an air-circulating oven. The dry films were removed and kept until the test in a tight bag. The film was given the CMC code using this command.

CMC/ CuO@ZnO nanocomposite films were produced by mixing various amounts of CuO@ZnO core/shell NPs (2 and 4 wt%) into the previously prepared CMC solution, as listed in Table 1. When the mixture turned grey after another 15 min of sonication at 50 °C, it was put into glass petri dishes and dried for 24 h in a 40 °C oven. Finally, the films were carefully taken out of the petri plates. The mean values of the thickness of three randomly chosen positions for each sample were calculated and are shown in Table 1, which were measured with a digital micrometer.

**Table 1:** Sample coding according to CuO@ZnO core/shell NPs concentration and the thickness of the nanocomposite films.

Structure	CMC (g)	CuO (g)	D.W. (mL)	Thickness (mm)
CMC	0.250	0.000	50	0.032
CS-1	0.245	0.005		0.024
CS-2	0.240	0.010		0.039

### Characterization techniques

XRD spectra in the range 25°–80° were obtained using a Philips X-ray diffractometer with a step size of 0.026° and a Cu-K1-2 source of = 1.54 A°, 45 kV, and 40 mA to analyze the crystalline structures of the generated nanocomposite samples of CMC and CuO@ZnO core/shell nanoparticles. Powdered XRD samples are obtained by hand grinding with a mortar and pestle (CuO@ZnO core/shell nanoparticles). Samples were typically crushed in a liquid medium such as ethanol to minimize sample loss during grinding and to mitigate structural damage to the phases in the sample caused by grinding. To learn more about the size and morphology of the produced CuO@ZnO core/shell nanoparticles, the nanoparticles was examined using high resolution—transmission electron microscopy (JEOL, JEM-2010-F) at a 200 kV accelerating voltage. The particles were dissolved in ethanol using a vortex mixer before being observed on a gold grid (300 mesh, TED PELLA, INC). The molecular structure of CMC and CuO@ZnO core/shell nanoparticles was investigated using Fourier transform infrared spectroscopy (Bruker vertex 70) in the wavenumber range (4000–400 cm^−1^). The diamond ATR accessory has a penetration depth of 2 m when used with a type II alpha diamond crystal. The spectra were recorded at a resolution of 4 cm^−1^. Each spectrum received 35 scans in total. With a resolution of 4 cm^−1^, the same settings were used to test the background versus air. The samples were used without preparation.

### Ethical approval

This work is not applicable for both human and/or animal studies.

## Results and discussion

### CuO@ZnO core/shell characterization

#### XRD characterization

**Figure 1:**
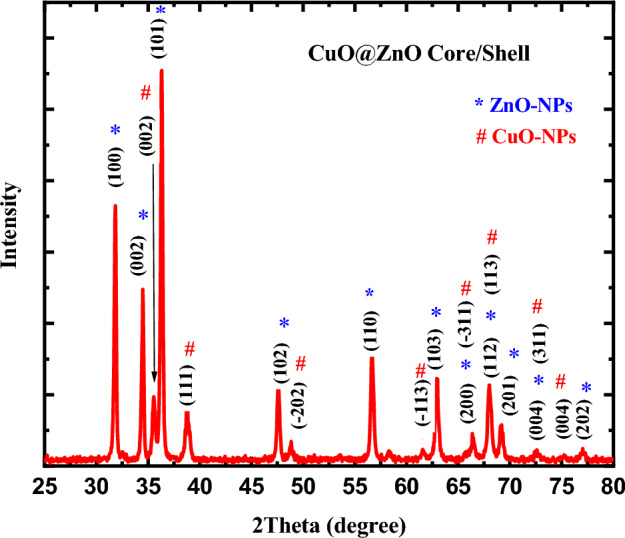
XRD pattern of synthesized CuO@ZnO core/shell nanoparticles.

The spectrum of XRD diffractogram of the prepared CuO@ZnO core/shell NPs was investigated as shown in Fig. 1. Eight peaks with high intensity were attributed to ZnO-NPs (code: 01-079-5604, space group P63mc (number 186), Hexagonal) as shown in the figure. From the figure, it is obvious that the prepared core/shell is crystalline in nature. As there were a number of remarkable peaks in the XRD pattern that appeared at 2theta = 31.70°, 34.35°, 35.44°, 36.18°, 38.67°, 47.47°, 48.68°, 56.54°, 61.54°, 62.80°, 66.21°, 67.89°, 69.02°, 72.44°, 75.12° and 76.84° which is indexed to the planes of (1 0 0), (0 0 2), (002), (1 0 1), (1 1 1), (1 0 2), (− 2 0 2), (1 1 0), (− 1 1 3), (1 0 3), (2 0 1), (0 0 4) and (2 0 2). Where, the planes (1 0 0), (0 0 2), (1 0 1), (1 0 2), (1 0 3), (2 0 0), (1 1 2), (2 0 1), and (2 0 2) corresponds to ZnO-NPs while, the planes of (0 0 2), (1 1 1), (− 2 0 2), and (0 0 4) corresponds to CuO-NPs.

However, the three positions of 2theta = 66.21°, 67.89°, and 72.44° corresponds to the planes of both CuO and ZnO which are superimposed on each other (planes (2 0 0), (1 1 2) and (0 0 4) for ZnO-NPs and (− 3 1 1), (1 1 3) and (3 1 1) for CuO-NPs). The strong diffraction peaks of ZnO, suggested that many ZnO-NPs were loaded on the CuO-NPs during the precipitation method. Moreover, the intensity of the CuO peaks in the XRD pattern of CuO@ZnO core/shell is lower than that of pristine CuO, which is due to the coating of ZnO-NPs on the surface of CuO-NPs. The peaks' sharpness indicates that the produced substance is crystalline^52^.

According to published research, there is a strong (002) diffraction peak with monoclinic structure at 35.44 in the XRD pattern of uncoated CuO nanoparticles (JCPDS 00-045-0937). This CuO structure, in which the oxygen and copper atoms are arranged in monoclinic orientations, is the most thermodynamically stable. As a result, following the development of the ZnO shell, CuO maintains its monoclinic unit cell-containing tenorite structure, as shown by the presence of the (002) diffraction peak in Fig. 1. The figure makes it abundantly clear that every conspicuous XRD peak agrees well with the monoclinic crystal structure of CuO and the wurtzite structure of ZnO. It is evident that the CuO@ZnO core/shell nanostructure is a two-phase substance made up of well-crystalline CuO and ZnO. Table 2 shows the lattice parameters of monoclinic CuO-NPs and hexagonal ZnO-NPs unit cell. The lack of additional peaks in the core/shell’s XRD pattern that could be attributed to secondary phases demonstrates the purity of the produced material.

**Table 2:** Comparison between calculated and JCPDS card’s lattice parameters for CuO@ZnO core/shell NPs.

CuO@ZnO core/shell NPs	Calculated values				JCPDS card			β (°)′
	a (A°)	b (A°)	c (A°)	β (°)	a (A°)	b (A°)	c (A°)	
Hexagonal ZnO-NPs	3.256	3.253	5.216	–	3.253	3.253	5.210	90
Monoclinic CuO-NPs	4.6965	3.4252	5.0613	100.113	4.685	3.425	5.130	99.549

For hexagonal and monoclinic unit cells, the ${d}_{hkl}$ spacing is related to the miller indices by the following two equations:

1 $$\frac{1}{{d}^{2}}= \frac{4}{3}\left(\frac{{h}^{2}+hk+{k}^{2}}{{a}^{2}}\right)+\frac{{l}^{2}}{c}$$

2 $$\frac{1}{{d}^{2}}= \frac{1}{{sin}^{2}\beta }\left(\frac{{h}^{2}}{{a}^{2}}+\frac{{k}^{2}{sin}^{2}\beta }{{b}^{2}}+\frac{{l}^{2}}{{c}^{2}}-\frac{2hl cos\beta }{ac}\right)$$

The Debye–Scherrer's formula which describes the link between crystal grain size and X-ray line broadening, has been used to quantify the average crystallite size of CuO@ZnO core/shell nanoparticles.

3 $$D=\frac{K\lambda }{\beta Cos\theta }$$

where, K is a parameter related to crystallite shape (K=1), λ, is the wavelength of the Cu-K_α_ radiation used in X-ray imaging (0.154 nm), β, is the full width at half maximum (FWHM) of the peaks, θ is the Bragg’s angle, and, D is the average crystalline size in nm. The calculated average crystallite size of the CuO@ZnO core/shell nanoparticles was thus determined to be 30.42 nm, using Scherrer's formula. Micro strain (ε), dislocation density (δ) and number of crystallites (N_c_) were also determined for the fundamental peak using the following equations.

4 $$\varepsilon =\frac{\beta cos\theta }{4}$$

5 $$\delta =\frac{1}{{D}^{2}}$$

6 $${\mathrm{N}}_{c}=\frac{t}{{D}^{3}}$$

where, t is the thickness of the sample. The calculated values of Micro strain (ε), dislocation density (δ) and number of crystallites (N_c_) of the CuO@ZnO core/shell nanoparticles were 3.67 × 10^–3^, 1.08 × 10^–3^ nm^−2^, and 355.08, respectively. The insignificant changes in the peak positions suggest that ZnO can modify CuO and alter its structure. The lattice deformation brought on by the Cu^+2^ and Zn^+2^ ions' different radii reduces the crystallite size. The strain field or stress that disrupts the process of grain formation is brought on by the varied sizes of radii. Smaller ionic radii increase the agglomeration and thus increase the crystallite size. and the ionic radius of Zn^+2^ is larger than that of Cu^+2^^53^.

#### HR-TEM characterization

**Figure 2:**
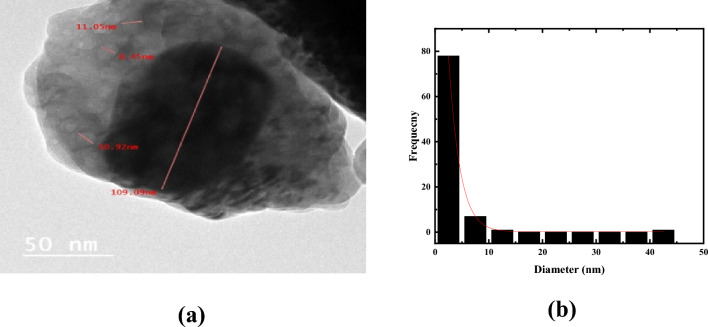
(**a**) HR-TEM image and (**b**) histogram of particle size distribution of synthesized CuO@ZnO core/shell nanoparticles.

Figure 2a depicts the HR-TEM images of CuO@ZnO core/shell NPs. The figure shows that the particles have a spherical shape and a narrow size distribution. The dark region reflects the CuO core and the surrounding gray layer reflects the ZnO shell. Thus, HR-TEM images confirm the formation of core/shell structure in the nanoscale. The presence of several small hexagonal nanoparticles formed above the CuO core structure confirm the XRD results that large number of ZnO NPs are coated on CuO during the Co-precipitation method. Meanwhile, Fig. 2b presents the histograms of particle size distribution of CuO@ZnO core/shell NPs. The figure show the size distribution of CuO@ZnO core/shell NPs, which is reasonably described by the Gaussian function, revealing a tight size distribution with average diameters of approximately 5.22 nm and a standard deviation of 0.18 nm.

#### FTIR characterization

**Figure 3:**
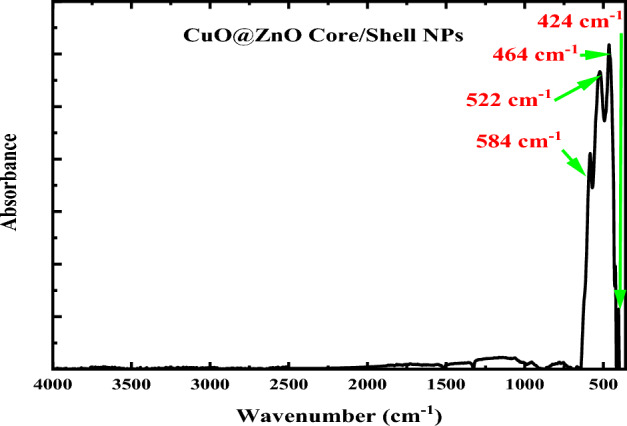
FTIR absorption spectrum of CuO@ZnO core/shell nanoparticles.

By using the FTIR approach, the functional groups included in the prepared CuO@ZnO core/shell nanoparticles have been identified. FTIR spectrum of the CuO@ZnO core/shell nanoparticles in the range of 4000–400 cm^−1^ is shown in Fig. 3. According to earlier FTIR spectroscopy results, interatomic vibrations cause the absorption bands in the fingerprint region, or below 1000 cm^−1^, that are present in metal–oxygen bonds^52^.

The combined impact of CuO and ZnO vibrations is what causes the strong absorption bands to appear in the region 400–600 cm^−1^. ZnO-NPs reportedly has an infrared absorption band at a wavelength of about 464 cm^−1^^54^. However, this value is not constant and is determined by the material's chemical composition, crystal structure, and particle morphology^55^. The FTIR absorption spectra of the CuO@ZnO core/shell nanoparticles consists of four absorption bands located at 584, 522, 464 and 424 cm^−1^ refer to the formation of metal–oxygen bonding. The ZnO-NPs absorption peak, which is associated with the stretching vibration of Zn–O, is visible in the current CuO@ZnO core/shell nanoparticles at 424 and 464 cm^−1^ which is in good agreement with that reported in the literature. The absorption band of ZnO-NPs that can be seen at about 464 cm^−1^ indicates that Zn (OH)_2_ had fully converted to ZnO-NPs. The presence of CuO, which represents Cu–O stretching, is indicated by the absorption bands at 584 and 522 cm^−1^^56^. As a result, FTIR data are consistent with those obtained from XRD and HR-TEM techniques, demonstrating the combination of CuO and ZnO in the synthesized core/shell with good stoichiometric composition. Moreover, the absence of absorption bands in the region of 4000 to 1000 cm^−1^ reflects the purity of the synthesized CuO@ZnO core/shell nanoparticles and that the calcination temperature was enough to obtain the NPs. Additionally, the absence of the OH group confirmed that all Cu(OH)_2_ and Zn(OH)_2_ were converted completely to CuO and ZnO^56^. This result confirms the XRD results, which confirmed the purity of synthesized CuO@ZnO core/shell nanoparticles due to the lack of additional peaks that could be attributed to secondary phases of CuO or ZnO NPs.

#### Optical results

**Figure 4:**
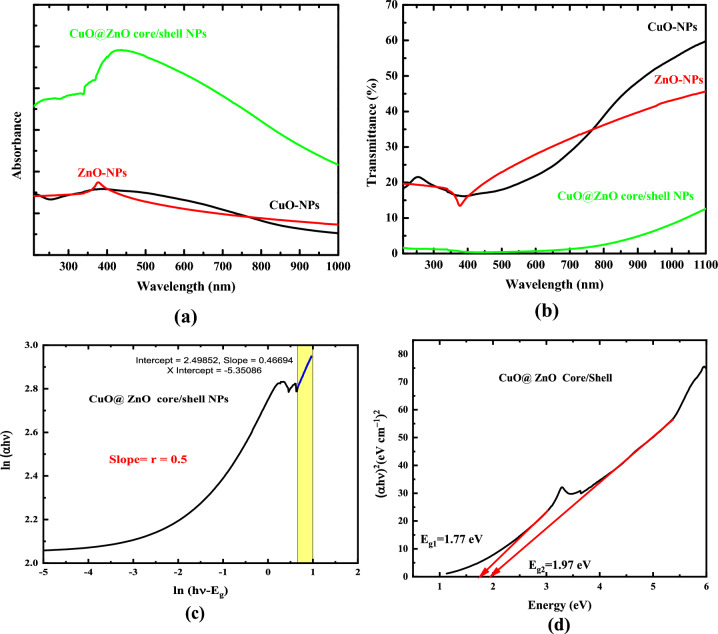
UV–Vis (**a**) absorption spectra of CuO, ZnO, and CuO@ZnO core/shell NPs, (**b**) transmittance spectra of CuO, ZnO, and CuO@ZnO core/shell NPs (**c**) variation of ln (αhν) with ln (hν–E_g_) to determine the type of transition, and (**d**) Direct allowed optical bandgap.

UV–Vis absorption measurements were performed on the produced nanoparticles to investigate the electronic interactions caused by the shelling of ZnO onto CuO nanoparticles. The absorption spectra of CuO@ZnO core/shell nanoparticles at room temperature in the wavelength range 200–1000 nm are shown in Fig. 4a. For nano-crystalline CuO@ZnO core/shell nanoparticles, the absorption spectra reveal two absorption peaks (located in the ultraviolet and visible regions), confirming the existence of CuO-NPs and ZnO-NPs.

Around 332 nm, the first notable peak that belonged to ZnO shell could be seen. Around 436 nm, a second broad peak appeared and was linked to the excitation of the surface Plasmon resonance of CuO nanoparticles (see Fig. 4a). The figure shows that the absorbance of pure CuO and ZnO NPs increased due to the formation of the ZnO shell for the same concentrations this means that the mechanism of CuO@ZnO core/shell nanoparticles filtration is the absorption of UV radiation. The figure showed that the band structure of CuO and ZnO NPs dramatically changed and differed when those two species successfully created a CuO@ZnO core/shell hybrid nanocomposite system via the formation of heterojunction. In comparison to pure CuO and ZnO NPs, CuO@ZnO core/shell NPs exhibited broad absorption extended from the UV region to both visible and infrared region. Additionally, Fig. 4b confirmed the highest capability of the synthesized core/shell NPs as a blocking material for UV light. It is clear that, for the same concentration, that the transmittance of CuO and ZnO NPs decreased due to the formation of the heterojunction between CuO and ZnO and that the core/shell NPs blocks nearly all the incident UV and visible wavelengths and extends to the near IR region.

Studying and learning more about the absorption spectra of the samples is vital in order to fully understand and gather data regarding the band structure of the materials. It is well known that the bandgap of a substance can be obtained using the fundamental absorption, that correlates to the electron transition from the valance band to the conduction band. Based on the Lambert–Beer law, absorbance, Abs, is related to the absorption coefficient, α, by the equation:

7 $$\alpha \, = { 2}.{3}0{3}*{\text{ Abs }}/{\text{ d}}$$

where, d is the sample thickness. Additionally, Tauc equation can be solved for the determination of the transition type by dividing it by the product of the differentiation, as shown by the following equation^51^:

8 $$\left( {\alpha {\text{h}}\nu } \right) \, = {\text{ B}}\left( {{\text{h}}\nu - {\text{E}}_{{\text{g}}} } \right)^{{\text{r}}}$$

9 $$\frac{d\left[ln\left(\alpha h\upsilon \right)\right]}{d[h\upsilon ]}=\frac{r}{(h\upsilon -{E}_{g})}$$

According to Tauc's law, the electron transitions of the materials normally rely on the energy of the incident photon. The formula was used to compute the connection between (αhν) and the energy of the incident photon (hν) in the region of strong absorption. B is the energy-independent band tail parameter and depends on the probability of electronic transition between the valance and conduction bands. r is the power parameter of transition type determined from the material’s nature, i.e., crystalline or amorphous. It can be 1/2, 2, 2/3, and 1/3 for direct allowed, indirect allowed, direct prohibited, and indirect prohibited transitions, respectively.

To determine the optical bandgap of CuO@ZnO core/shell nanoparticles we need to determine the type of transition firstly. Accordingly, the dependence of ln (αhν) on ln (hν–Eg) for CuO@ZnO core/shell NPs is determined as presented in Fig. 4c. Plotting ln (αhν) against ln(hν–Eg) will reveal the value of r. As a result, the slope of the figure gives the value of r almost equaling 0.5 in Eq. (9). According to this presumption, the direct transitions that are permitted form the fundamental shoulder of absorption for CuO@ZnO core/shell NPs.

The band structure of CuO and ZnO NPs dramatically changed and differed when those two species successfully created a CuO@ZnO core/shell hybrid nanocomposite system via the formation of heterojunction. In comparison to pure CuO, CuO@ZnO core/shell NPs had an estimated band gap of 1.77 and 1.97 eV corresponds to the two transitions and exhibited broad absorption extended from the visible region to the infrared range (see Fig. 4d). Simultaneously, after ZnO coating, the composites improved absorption strength in all regions of the electromagnetic spectrum which was most likely due to hetero-junction between the p-CuO core and the n-ZnO shell, as well as defects introduced during the synthetic process^57^.

**Figure 5:**
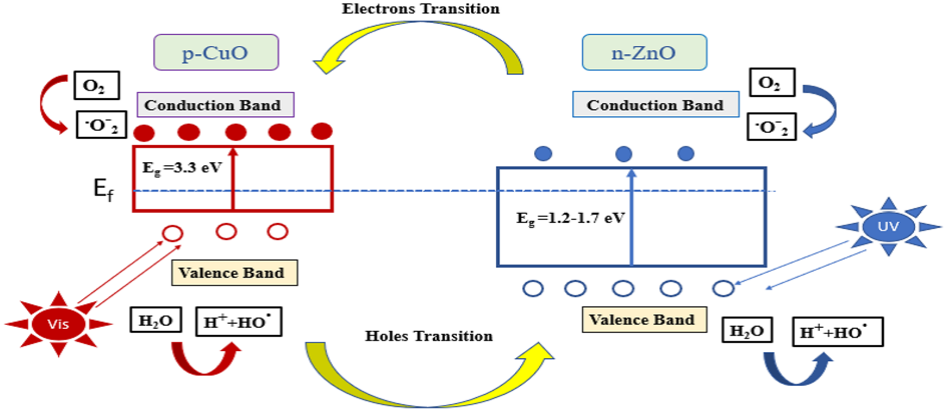
The supposed mechanism for charge carriers’ transportation through CuO@ZnO core/shell NPs.

The following section discusses the high activity of the produced CuO@ZnO core/shell NPs. The ZnO shell might be permeable to sunlight, allowing the CuO core to be directly driven by it. When exposed to sunshine, ZnO and CuO produced electrons (e^−^) and holes (h^+^) (Fig. 5), respectively. The produced e^-^ in the ZnO shell's conduction band (CB) is transferred to the CuO core's CB. This is because the CB of ZnO is higher than that of CuO, allowing Cu^2+^ to take electrons from Zn^+2^ and create Zn^+^. Furthermore, the CuO valence band (VB) is more negative than that of ZnO, indicating that holes are transferred from CuO to ZnO^58^.

### CMC/CuO@ZnO core/shell nanocomposite films characterization

#### XRD results

**Figure 6:**
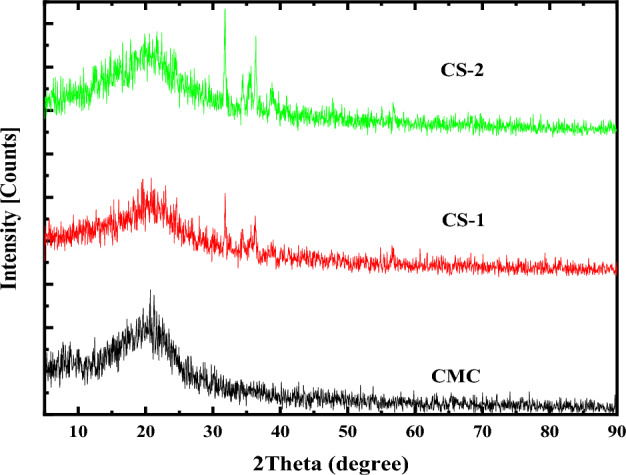
XRD of pure CMC and CMC doped with 2 and 4 wt% of CuO@ZnO core/shell nanoparticles.

Figure 6 presents the XRD pattern of the virgin CMC and CMC doped with 2 and 4 wt% of CuO@ZnO core/shell NPs over the 2θ range of 5° to 90°. Based on the obtained XRD pattern of virgin CMC a broad peak is noticed at 20°, reflecting CMC's amorphous nature. The addition of 2 wt% of CuO@ZnO core /shell (denoted by CS-1) results in several peaks centered at 31.77°, 34.36°, 35.55°, 36.25° and 56.62°. The peak at 35.55° refers to the Miller indices of the reflecting plane (002) of CuO core NPs. Meanwhile, the peaks observed at 31.77°, 34.36°, 36.25° and 56.62° refers to the planes: (100), (002), (101), and (110) for ZnO shell. These peaks are corresponded to the standard JCPDS cards no. 01-078-3315 and 01-078-3315 for CuO and ZnO, respectively. Similar results are observed in the XRD pattern of the film containing 4 wt% of CuO@ZnO core /shell NPs (denoted by CS-2) with the appearance of additional peaks. The XRD peaks observed for the sample abbreviated as CS-2 are 31.93°, 34.61°, 35.64°, 36.37°, 38.87°, and 56.81° corresponds to the planes (1 0 0), (0 0 2), (0 0 2), (1 0 1), (1 1 1), and (1 1 0). No additional XRD peaks related to other phases of CuO or any other impurities are detected in the XRD pattern compared with that of the prepared CuO@ZnO core/shell NPs. This result confirms the success of synthesizing hybrid nanocomposite films based on CMC and CuO@ZnO core/shell NPs.

Additionally, strong reflections appeared in Fig. 6 along the (100) plane of ZnO-NPs and (200) plane of CuO-NPs. Moreover, the intensity of the XRD peaks along these planes decreases as the CuO@ZnO core/shell concentration in the host matrix increases. The decrease in the peak intensity refers to a decrease in the crystallinity of the films.

Doping a large ionic radius element (Zn^2+^) into the sites of a smaller one (CuO^2+^) is thought to be the cause of the decrease in crystallinity, which is followed by a relative increase in defects and randomness. Furthermore, as shown in the figure for all peaks, there are noticeable shifts in the XRD peaks of the doped CMC films with increasing core/shell concentration. This behavior demonstrates a difference in the doped samples' crystalline structure parameters^27^.

Additionally, using the aforementioned related equations (Eq. (3) to Eq. (6)) and the obtained (0 0 1) Miller's plane data, the crystallite size (D), micro-strain (ε), dislocation density (δ) and the number of crystallites (Nc) of the prepared films have been estimated. These structures’ parameters are listed in Table 3. The obtained crystallite size (D) decreased to 41.60 nm (sample CS-2). The primary causes of this variation in D are the lattice distortion and internal stress brought on by the ZnO shell^59^. Moreover, the decrease in the intensity of the hybrid nanocomposite compared to the pure CMC, which was used to confirm the increases in defect density, may be a key factor in D variations. The micro strain and the dislocation density exhibit the opposite behavior, as presented in Table 3.

**Table 3:** Crystallite Size (D), dislocation density (δ), micro strain (ϵ) and the number of crystallites (Nc) of the most prominent peak [i.e., plane (100)] in CMC/CuO@ZnO core/shell hybrid nanocomposite films.

Structure	D (nm)	δ (nm^−2^) × 10^–3^	ϵ × 10^–3^	Nc
CS-1	47.46	0.44	2.68	22.45
CS-2	41.60	0.58	20.09	54.16

#### FTIR characterization

**Figure 7:**
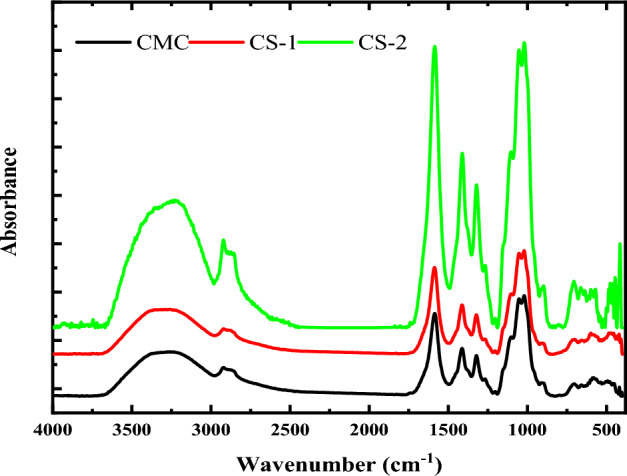
FTIR spectra of virgin CMC and CMC doped with 2 and 4 wt% of CuO@ZnO core/shell nanoparticles.

Hydrogen bond formation in the CMC hybrid nanocomposites can be explained by IR spectroscopy since hydrogen bonding changes the stretching vibration frequencies^60^. The FTIR absorbance spectra of the virgin CMC and its nanocomposites are shown in Fig. 7. Many significant absorption bands have been found in the FTIR absorption spectrum of the pure CMC and CMC doped with 2 and 4 wt% of CuO@ZnO core/shell NPs (denoted by CS-1 and CS-2, respectively), and they can be interpreted as follows: The characteristic absorption bands of pure CMC was similar to that observed in our previous work^51^. The CMC's vibration bands were successively centered at 3261, 2919, 1585, 1412, 1322, 1052, 1019, 912, and 657–534 cm^−1^ for the vibrations of OH stretching, CH stretching, COO^-^ asymmetric stretching, CH_2_ scissoring, OH bending, C–O–C bending, C–O bond of the CH_2_ OH, C–O stretching with some CH_2_ rocking motion, and ring stretching and deformation, respectively.

There are significant differences in the FTIR spectra of CMC/CuO@ZnO hybrid nanocomposite films. The centers and intensities of the absorption bands are among the changes. Adding 2 and 4 wt% of CuO@ZnO core/shell NPs results in a strong shift of the absorption band assigned to OH stretching to 3292 and 3238 cm^−1^, respectively (see Table 4). This evidence supports the active interaction of the core/shell NPs with the host polymer. Furthermore, as the core/shell NPs concentration in the host polymer is increased to 4 wt%, the absorption peak shifts to shorter wavenumbers.

**Table 4:** FTIR band assignment of virgin CMC and CMC filled with 2 and 4 wt% of CuO@ZnO core/shell NPs.

Wavenumber (cm^−1^)			Band assignment
CMC	CS-1	CS-2	
3261	3292	3226	Stretching of OH group
2919	2922	2919	CH stretching of the CH_2_ groups
-	-	2892	CH asymmetric stretching
1585	1587	1585	Carboxylate group (COO^−^) asymmetric stretching
1412	1413	1411	CH_2_ scissoring
1322	1321	1321	OH bending
1052	1051	1051	The C–O–C bending vibration
1019	1020	1018	C–O bond of the CH_2_ OH group
912	912	912	C–O stretching + CH_2_ rocking motion
–	538	570	O–Cu–O
–	516	518	O–Cu–O
–	433	474	O–Zn–O
–	420	420	O–Zn–O

The presence of the bound OH group may cause the absorption band to shift to a lower frequency. As a result, the intensity will rise, and an asymmetrical band will be observed at 2892 cm^−1^. Along with that, the band will be expanded. The strength and interactions of the hydrogen bond in the polymer matrix are measured by hydroxyl absorption^61^.

Also, Fig. 7 confirmed the existence of the characteristic absorption bands of CuO@ZnO core/shell NPs. The absorption bands located at 538 and 516 cm^−1^ are attributed to the stretching of Cu–O bond. Meanwhile, the ZnO characteristic bands are observed at 433 and 420 cm^−1^. Increasing the core/shell concentration shifts these bands to the higher wavenumber region as presented in Table 4. These bands confirmed the combination of the CMC characteristics with that of the CuO@ZnO core/shell NPs inside the hybrid nanocomposite. In addition, reasonable shift in the CuO@ZnO core/shell NPs bands in the nanocomposite spectra is observed that is due to the interaction between CuO@ZnO core/shell NPs and the OH group of CMC. The absence of any secondary phases of CuO confirmed the XRD results.

#### Optical results

##### Determination of absorption parameters

**Figure 8:**
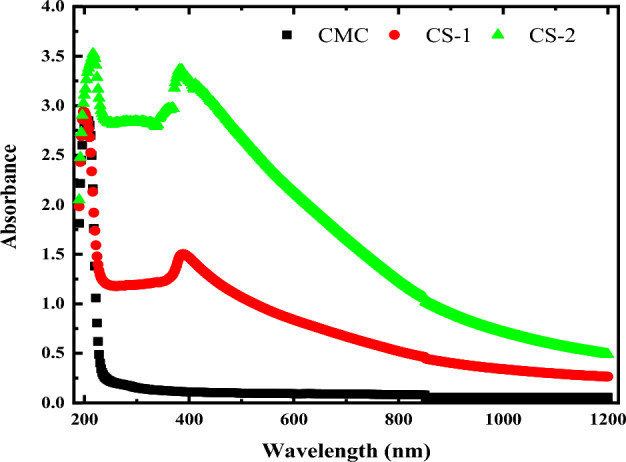
The optical absorbance for pure CMC and CMC doped with 2 and 4 wt% of CuO@ZnO core/shell nanoparticles.

Investigation of the optical properties of materials is a useful tool for examining the band structure and the density of the electronic state. When polymer mixtures are combined with filler, their optical properties change frequently^37^. Figure 8 shows the normal optical absorption of pristine and hybrid samples in the 200–1200 nm spectral range. On the other hand, it was seen in the figure that the absorption edge of CMC was red shifted due to the addition of CuO@ZnO core/shell NPs, moving to a longer wavelength (from 208 to 216 nm) which corresponds to the π–π* transition of CMC. This red shift behavior was predicted to result in a smaller optical bandgap (E_g_) value. The aggregation in the samples may cause this redshift^63^. The observed shift refers to the strong interaction between CMC functional groups and CuO@ZnO core/shell NPs. Additionally, the characteristic absorption peak of the CuO@ZnO core/shell appeared with high intensity in the absorption spectra of CMC/CuO@ZnO hybrid nanocomposite films at 388 nm for the samples containing 2 and 4 wt% of CuO@ZnO core/shell NPs. This confirms the formation of the hybrid nanocomposite and the formation of hydrogen bonding between CMC and CuO@ZnO core/shell NPs.

**Figure 9:**
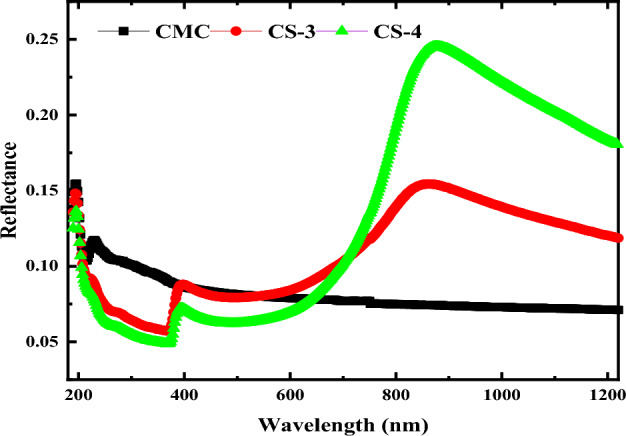
The optical reflectance R(λ) for pure CMC and CMC doped with 2 and 4 wt% of CuO@ZnO core/shell nanoparticles.

The spectral variation of reflectance with wavelength for pure CMC and CMC/CuO@ZnO core/shell nanocomposite films are presented in Fig. 9. Furthermore, the reflectance of the pure CMC was increased as it filled with 2 and 4 wt% CuO@ZnO core/shell NPs, reaching its highest value as it loaded with 4 wt% CuO@ZnO core/shell NPs. At *λ* = 750 nm, the reflectance of pure CMC is 7.7% and it increased to 11.9 and 13.5% as it loaded with 2 and 4 wt% CuO@ZnO core/shell NPs, respectively. This modification in the reflectance data upon filling CMC with 2 and 4 wt% CuO@ZnO core/shell NPs may be caused by the alteration in the packing density of the host polymer^62^. The high reflectivity of CMC/CuO@ZnO nanocomposite films nominated it to use in the manufacture of marking paint, reflective tape, leather, cloth, and safety silk fabric**.**

The absorption coefficient is an important parameter for evaluating the fluctuation of the band structure of polymer materials. It provides important information regarding the nature of the forbidden photonic gap energy, which will be used in all future applications. For accurate results the absorption coefficient can be calculated using the following equation:

10 $$\alpha =\frac{1}{d}\mathrm{ ln }[({\left(1-R\right)}^{2}/2T)+\sqrt{\left(\frac{{\left(1-R\right)}^{4}}{4{T}^{2}}\right)+{R}^{2}}]$$

**Figure 10:**
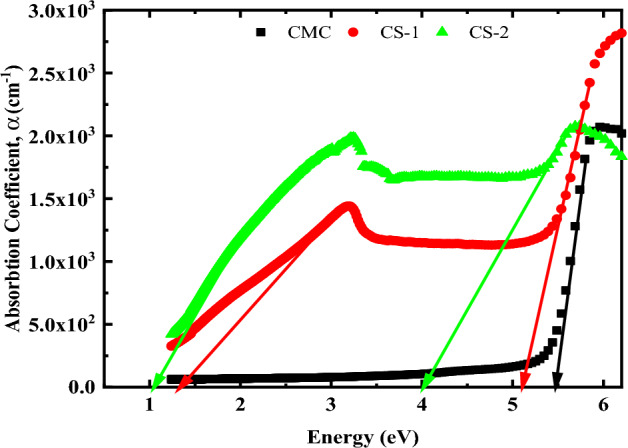
The optical absorption coefficient (α) for pure CMC and CMC doped with 2 and 4 wt% of CuO@ZnO core/shell nanoparticles.

This is due to the non-accurate measurements of the sample thickness^63^. Figure 10 presents the variation of the absorption coefficient with the incident energy. It is clear from the figure that the value of α of pure CMC increases after the addition of CuO@ZnO core/shell NPs. This confirms the important interactions between the CMC and CuO@ZnO core/shell NPs matrices. The value of the fundamental edge is obtained by extrapolating the linear component of the curve to the energy axis for all samples. Two absorption edges were observed in the figure indicating the presence of two optical band gaps. Additionally, it’s clear that the absorption edge of pure CMC shifted to the lower energy region by increasing CuO@ZnO core/shell NPs concentration. This result matches the XRD and FTIR performance of the nanocomposite films because of the percentage of NPs on the microstructure of the CMC matrix. Figure 10 also shows that as photon energy increases, the absorption coefficient gradually increases, resulting in a plateau. Such an increase is common in indirect bandgap semiconductors^64^. The shifts in the absorption edge towards lower photon energy for the nanocomposite samples indicate that the optical bandgap has shrunk. This large shift in the absorption edge can be explained by the development of intramolecular charge transfer (ICT) in the host CMC polymer, resulting in an increase in intermolecular stacking^65^.

##### Optical band gap determination

**Figure 11:**
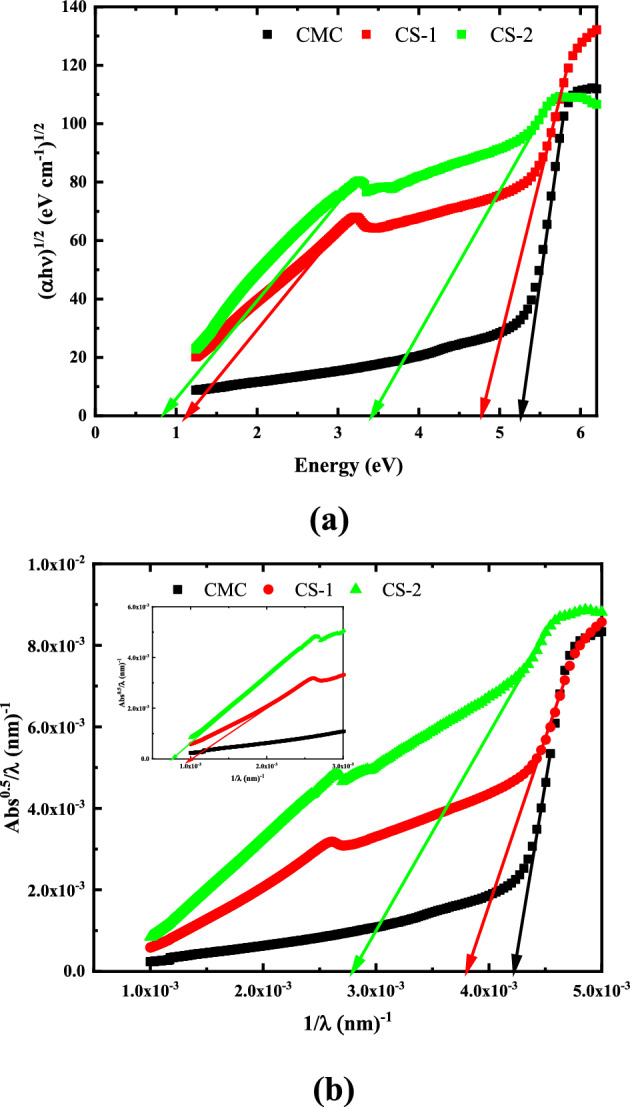
Indirect allowed optical bandgap energy determination using: (**a**) Tauc model and (**b**) ASF model for pure CMC and CMC doped with 2 and 4 wt.% of CuO@ZnO core/shell NPs.

Based on our previous work it is found that Pure CMC has an indirect optical bandgap^51^. Accordingly, Fig. 11a depicts the plot of (αhν)^1/2^ versus photon energy (hν) for CMC/CuO@ZnO core/shell nanocomposite films. It has been established that the top of the valance band and the bottom of the conduction band are located at the same zero crystal momentum (i.e., wave vector or k-vector) in direct bandgap materials and at different K values in indirect bandgap ones^51^. Because of the momentum conservation law, indirect band gap semiconductors are good candidates for UV-Blocking applications. In indirect semiconductors, the momenta of holes and electrons differ. Thus, in order to recombine and satisfy the momentum conservation equation, they must do something with the uncompensated momentum. In direct band gap semiconductors, electron and hole–pair momentum can be zero, allowing them to recombine. It is worth noting that the indirect optical bandgap (HOMO/LUMO optical bandgap) decreased from 5.27 eV for pure CMC to 3.43 eV for CMC doped with 4 wt% CuO@ZnO core/shell NPs. Additionally, the figure shows that the nanocomposites samples possess another absorption edge, located in the low energy region, that corresponds to the Onset band gap. The bandgap values for all nanocomposites are tabulated in Table 5. The optical bandgap (E_g_) of the doped samples was found to be smaller. Such a decrease can be attributed to the formation of intramolecular charge transfer in the host polymer as a result of the incorporation of trace amounts of fillers and to the changes in the disordering degree in the CMC matrix. This behavior reflects the complexation between the oxygen atom of the CuO@ZnO core/shell NPs and the OH group of CMC. As a result, lower energy transitions will be enhanced, resulting in observable changes in optical bandgap. Shifting to the visible region and observing the additional absorption peaks of the doped films confirms this viewpoint. Previous research has established the importance of organic polymers, functional materials, and composites with small optical bandgaps for photonics, organic light-emitting diode (OLED), and optoelectronics device applications.

**Table 5:** Indirect allowed optical bandgap energy obtained by using Tauc and ASF models and Urbach energy for pure CMC and CMC filled with 2 and 4 wt% CuO@ZnO core/shell NPs.

Structure	${\mathrm{E}}_{\mathrm{g}1}^{\mathrm{in}}$(eV)	${\mathrm{E}}_{\mathrm{g}2}^{\mathrm{in}}$(eV)	${\mathrm{E}}_{\mathrm{Opt }1}^{\mathrm{ASF}}$(eV)	${\mathrm{E}}_{\mathrm{Opt }2}^{\mathrm{ASF}}$(eV)	Eu (eV)
Pure CMC	5.27	–	5.24	–	0.21
CS-1	4.78	1.13	4.72	1.23	1.82
CS-2	3.43	0.83	3.46	0.89	2.04

The absorption spectrum fitting (ASF) model was applied to confirm the optical bandgap energy determined using Tauc equation for the nanocomposites under study, utilizing the UV absorption spectra measured (see Fig. 11b)^66^. The ability to calculate the optical gap energy (${E}_{Opt}^{ASF}$) of the samples without having to assess their thickness distinguished this method. The calculation is solely based on the samples' absorbance data. According to Souri and Shomalian, the optical gap energy depends on the wavelength of the incident photon and is expressed as follows:

11 $${E}_{Opt}^{ASF}= \frac{1240}{{\lambda }_{Opt}^{ASF}}$$

According to the ASF model, the E_g_ values of CMC/CuO@ZnO core/shell nanocomposites decreased with increasing CuO@ZnO core/shell NPs concentration strongly. These values are listed in Table 5. The results showed that the two models are consistent with each other.

Several solid-state devices (integrated optical circuits, emissive displays, optical sensors, etc.) can have their performance improved by adding a high refractive index coating to the sensing surface of the device (a regular change from the high refractive index of the active circuitry to the low index of air permits light to be coupled more efficiently). The refractive index of the materials changed when the energy gap changed, which is affected by localized states in the prohibited band. The absorption coefficient variation contributed to the band tail (Urbach energy). The tail energy (E_u_) value denotes the presence of flaws and disorder in the polymer matrix. It is found near the valence and conduction bands' borders inside the forbidden bandgap.

**Figure 12:**
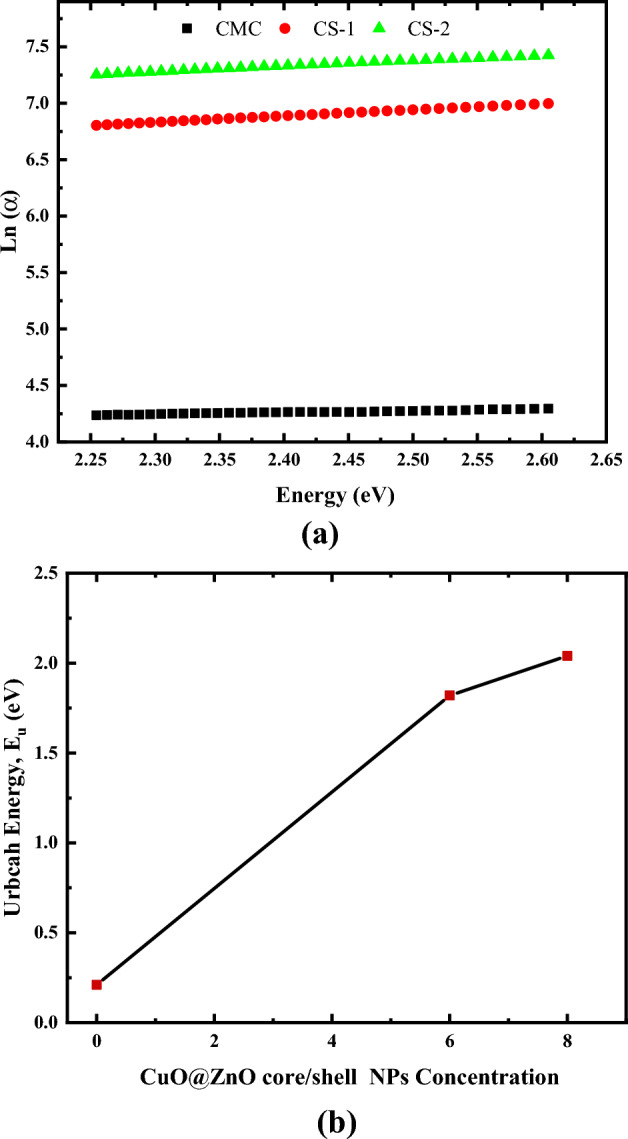
Variation of: (**a**) Ln α with photon energy, (**b**) Urbach energy with CuO@ZnO core/shell NPs concentration.

The values of E_u_ are evaluated for all films using the following equation^66^:

12 $$\alpha ={\alpha }_{o}\mathrm{exp}\left(h\upsilon /{E}_{u}\right)$$

where, the normalized transition strength is ${\alpha }_{o}$, and the Urbach energy is E_u_. As shown in Fig. 12a, Urbach tail energy (E_u_) was computed by graphing the logarithm values of the absorption coefficient (ln α) with the photon energy (hν). The E_u_ values are calculated by fitting the straight part of the graph to the inverse of its slope.

As described previously, the compositional differences of E_u_ are related to the nature and content of defects/disorders formed in the prohibited gap. These findings are consistent with the findings of the structural study. As shown in Fig. 12b, the E_u_ values for the CMC/CuO@ZnO core/shell nanocomposite films; it ranges between 0.21 and 2.04 eV (see Table 5). This value is greater than that of CMC loaded with 4 wt% of CuO- NPs in our previous work^51^.

##### Determination of dispersion parameters of CMC and CMC /CuO@ZnO hybrid nanocomposite films

The refractive index (n) and extinction coefficient (k) are calculated from the reflectance (R) of the film using the following two equations^19^:

13 $${\text{n }} = \, \left[ {\left( {{1} + {\text{R}}^{{{1}/{2}}} } \right)/ \, \left( {{1} - {\text{R}}^{{{1}/{2}}} } \right)} \right]$$

14 $${\text{k }} = \, \alpha \lambda /{4}\pi$$

**Figure 13:**
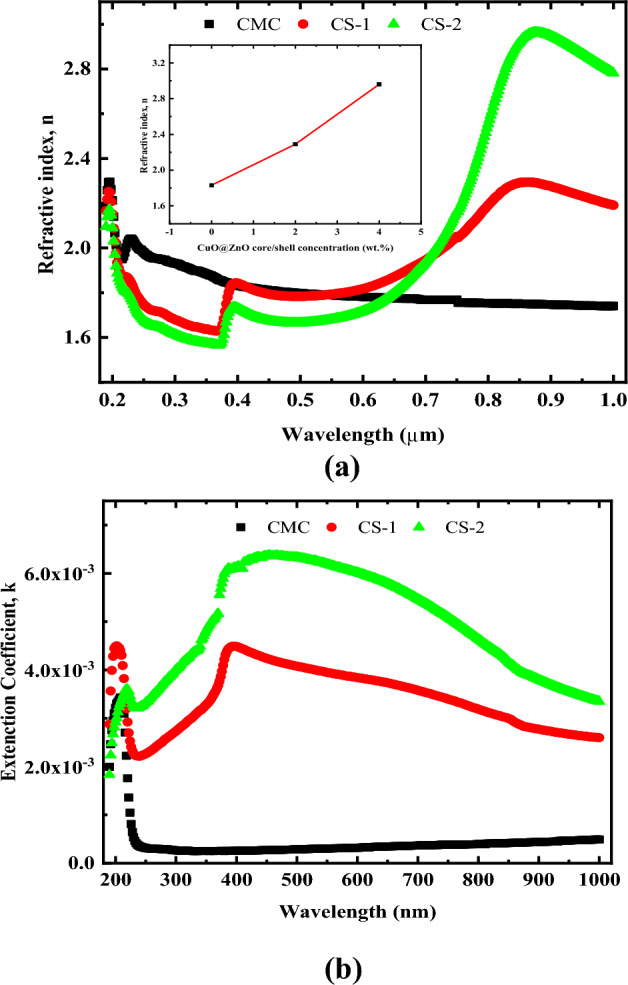
Variation of (**a**) refractive index n, and (**b**) extinction coefficient k as a function of wavelength for pure CMC and CMC doped with 2 and 4 wt% CuO@ZnO core/shell NPs.

Figure 13 depicts the variation of refractive index and extinction coefficient of CMC/CuO@ZnO hybrid nanocomposite films as a function of wavelength. It is important to note that the dispersion behavior of the nanocomposite samples varies with wavelength, whereas the pure CMC sample has an inverse relationship with wavelength. Because they are absent in the pure CMC refractive index spectra, the obvious peaks in the Near infrared region could be because of the CuO@ZnO core/shell NPs.

The changes occurred in the electronic structure of the doped samples can be linked to the increase in refractive index from 1.83 to 2.96 and it’s shifting. The inset of Fig. 13a depicts the samples' refractive index as a function of CuO@ZnO core/shell NPs concentration. This improvement can be explained by polarizability and density. With the addition of CuO@ZnO core/shell NPs, more charge carriers (dipole molecules) are introduced into the CMC matrix. As a result, there are more polarizable molecules, and higher densities result in a higher refractive index according to the well-known Lorentz–Lorenz formula.

The spectral behavior of the refractive index could also be divided into two distinct regions. The anomalous dispersion region lies between 250 and 400 nm. In this region, incident photons resonate at the same frequency with the film's surface plasma, revealing strong electron coupling in the nanocomposite matrix^19^. The normal dispersion region, on the other hand, is where 600 > λ > 400 nm. The refractive index decreases continuously as the wavelength increases in this region. However, beyond 600 nm the refractive index increases sharply, and this can be attributed to the free carrier contribution.

The refractive index at a higher wavelength is linearly proportional to the filler concentration, as presented in the inset of Fig. 13a. This means that the nanofillers are well distributed throughout the CMC matrix. This particle distribution processing within the host CMC can be viewed as an alternative method for high resolution transmission electron microscopy (HR-TEM) investigations. From the preceding discussion, understanding the refractive index is critical for both particle distribution and optical device design.

The spectra of k at different CuO@ZnO core/shell NPs concentrations as a function of incident wavelength are shown in Fig. 13b. Significant increase in k values was observed indicates that the electromagnetic waves in the visible range pass through the films with significant decay or inhibition. Furthermore, as the concentration of CuO@ZnO core/shell NPs in CMC matrix increases, the k value increased. As a result, when compared to the behavior of pure CMC films, CMC/CuO@ZnO hybrid nanocomposite films have a significant ability to attenuate incident light. These findings offer valuable insights into the superior optical applications of the studied polymeric films based on CMC/CuO@ZnO core/shell hybrid nanocomposites.

Furthermore, the optical dispersion parameters are the most significant parameters in the area of optoelectronic applications. These parameters are computed using the Wemple-Di Domenico (WDD) single-oscillator model, which is denoted by:

15 $$\frac{1}{({n}^{2}-1)}=\frac{{E}_{o}}{{E}_{d}}-\frac{{E}^{2}}{{E}_{d}{E}_{o}}$$

where, E_d_, is the dispersion energy, and E_0_, is the single oscillator energy^67^. To gain a better understanding of the physical properties of the samples being studied, further information about some parameters such as the lattice dielectric constant ${\varepsilon }_{l}$ and carrier concentration N is required. The real component of the dielectric constant ${\varepsilon }_{l}$ is related to λ^2^ according to Eq. (16):

16 $${n}^{2}={\varepsilon }_{l}-({e}^{2}N/4{\pi }^{2}{\varepsilon }_{o}{m}^{*}{c}^{2}) {\lambda }^{2}$$

**Figure 14:**
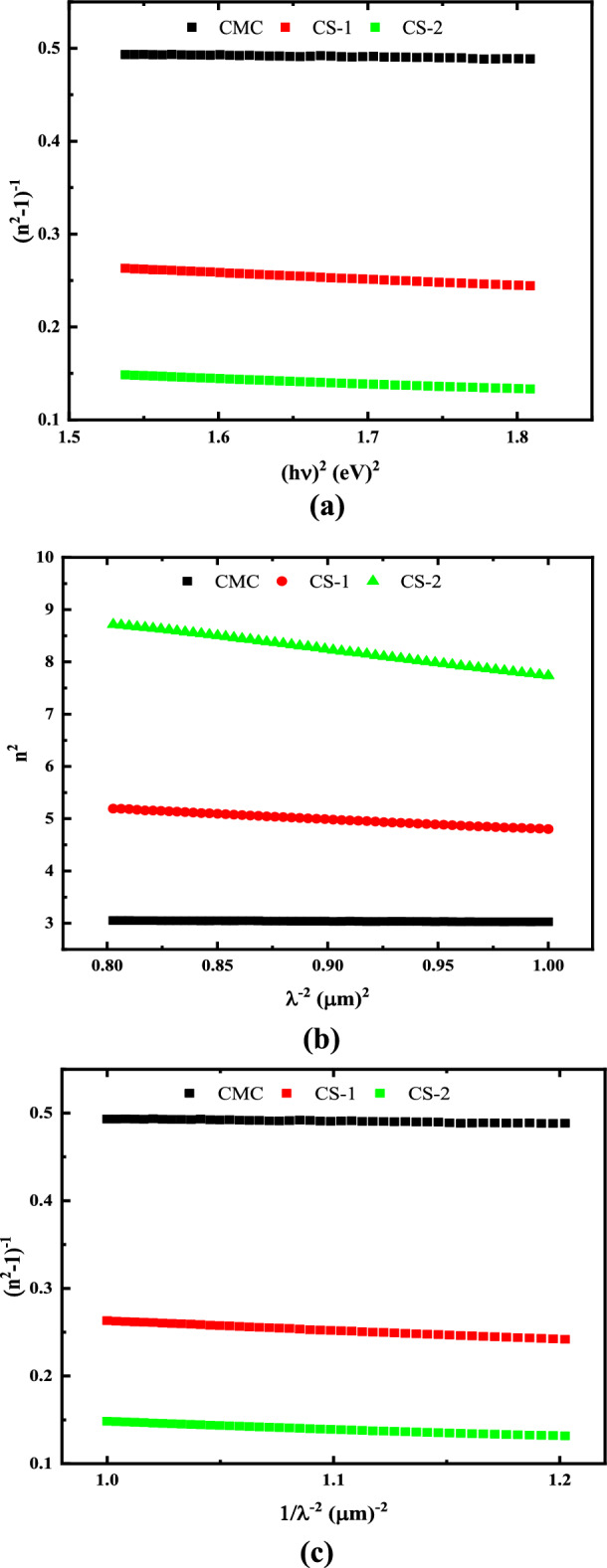
Optical dispersion parameters for pure CMC and CMC doped with 2 and 4 wt% CuO@ZnO core/shell NPs.

Figure 14a–c depicts the relationship between (n^2^ − 1)^−1^ and (hν)^2^, n^2^ with λ^−2^, and (n^2^ − 1)^−1^ with λ^−2^, respectively for wavelengths greater than 400 nm. This relationship is represented by a straight line. These lines intersect the (n^2^ − 1)^−1^ axis at (E_0_/E_d_) points and has a slope equal to (1/E_0_E_d_). Table 6 displays the calculated values of all dispersion parameters. Table 6 shows that, the values of E_0_ decrease and E_d_, ${\varepsilon }_{o}$, ${\varepsilon }_{l}$, N/m* and N increase as the concentration of CuO@ZnO core/shell NPs in the CMC matrix increases. This indicates an increase in charge transfer between the polymer macromolecules and the core/shell NPs, as well as an increase in the degree of disordering in the polymer structure. The obtained results confirmed the structural investigation of XRD and FTIR.

**Table 6:** Optical dispersion parameters of pure CMC and CMC with different concentration of CuO@ZnO core/shell NPs.

CuO@ZnO wt%	Absorption parameter		Dispersion parameter					
	Onset gap (eV)	HOMO–LUMO gap (eV)	E_O_ (eV)	E_d_ (eV)	${\varepsilon }_{\infty }$	${\varepsilon }_{l}$	N/M* (g^−1^ cm^−3^)	N (g^−1^ cm^3^)
0	–	5.27	5.00	9.32	3.01	3.16	4.41 × 10^55^	4.02 × 10^28^
2	1.13	4.78	2.28	6.10	3.65	6.60	5.69 × 10^56^	5.18 × 10^29^
4	0.83	3.43	2.08	8.50	5.13	15.65	1.56 × 10^57^	1.42 × 10^30^

##### UV-blocking capacity of CMC/CuO@ZnO hybrid nanocomposite films

**Figure 15:**
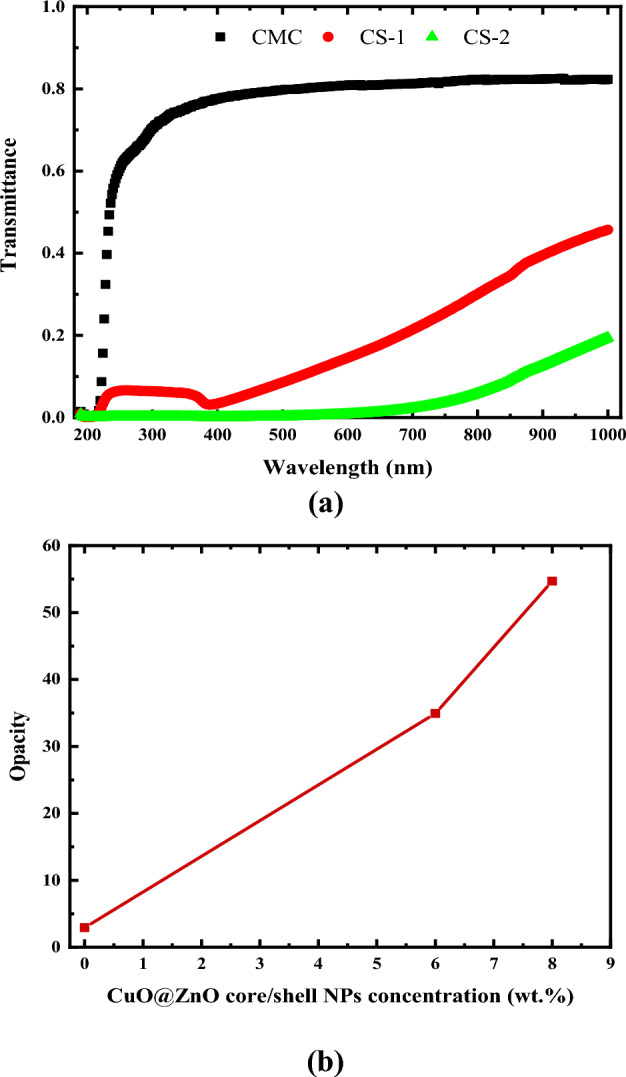
CMC/CuO@ZnO hybrid nanocomposite: (**a**) optical transmittance (T) as a function of wavelength (λ), and (**b**) opacity as a function of CuO@ZnO NPs concentration.

The UV–Vis. transmittance spectra of pure CMC film and CMC/CuO@ZnO hybrid nanocomposite films with varying CuO@ZnO core/shell content are shown in Fig. 15a. The figure shows that the pure CMC film lacks UV-shielding properties. The transmittance of CMC/CuO@ZnO hybrid nanocomposite films in the UV (200–400 nm) and visible regions (400–800 nm) decreases significantly when CuO@ZnO core/shell NPs are added. When the CuO@ZnO core/shell NPs content increases to 4 wt%, approximately 99.9% of UV light is absorbed. These findings confirmed that CuO@ZnO core/shell NPs had a high UV absorption capacity. As the transmission in UVC nearly equal 0, transmission in UVB remains constant at 0.1%, and transmission in UVA decreased to 0.05%. Accordingly, it can be concluded that the CMC/CuO@ZnO core/shell hybrid nanocomposite films can block approximately 100% of UVR.

Figure 15b shows the relationship between opacity and CuO@ZnO core/shell NPs concentration for pure CMC and nanocomposite films at 600 nm. The opacity of the films was calculated using Eq. (17) ^51^:

17 $$Opacity= \frac{{A}_{600}}{d}$$

where, A_600_ is the film absorbance wavelength of 600 nm and d is the film thickness (mm).

The results showed that the sample CS-2 has the higher opacity value as presented in Fig. 15b. Table 7 presents the increased values of the UV blocking percentages with increasing CuO@ZnO core/shell NPs concentration. It’s clear that adding 4 wt% of CuO@ZnO core/shell NPs to the CMC matrix results in higher UV blocking percentage than that obtained by adding 8 wt% of CuO-NPs reported in the literature^51^. This means that the formation of CuO@ZnO core/shell NPs reduces the cost and increased the efficiency. Thus, the CMC/CuO@ZnO core/shell hybrid nanocomposites can be used as a blocking material in skin care products.

**Table 7:** UV blocking percentages in the UVC, UVB, and UVA regions for pure CMC film and CMC doped with different concentrations of CuO@ZnO core/shell NPs.

CMC loaded with CuO@ZnO core/shell (wt%)	UV-blocking %		
	UVC	UVB	UVA
0	34%	27–34%	23–27%
2	94%	94%	94–97%
4	100%	100%	100%

Table 8 compares previous work on the UV blocking capabilities of various transparent polymer films, including the current study. UV blocking without the use of nanoparticles such as ZnO or TiO_2_ appears to be difficult, and only a few organic compatible systems exist, such as furan-based polyesters or modified dopamine-containing polymers**.**

**Table 8:** UV blocking properties of different polymeric systems reported in literature.

Polymer	Additive	% Transmittance			Comments (best performance)	Reference
		UVC	UVB	UVA		Reference
PVA	Dopamine − Melanin NP	0	0	10–70%	5 vol% NP	^68^
PVA	Carbon quantum dots	0	10%	5–60%	1 wt.% Carbon	^69^
PMMA	ZnO quantum dots	0	0	40–50%	0.05 wt.% ZnO	^70^
Waterborne acrylic	TiO_2_–Al_2_O_3_–POSS	0	0	60%	POSS	^71^
Bifuran polyester	Furan-based dicarboxylic acids	0	0	5%	Furan blocks UV	^72^
PVA	Wood nanofibers	0	5–10%	80%	10 wt% nanofibers	^73^
Fish gelatin	ZnO nanorods	0	0	3–15%	5 wt% ZnO	^74^
Carrageenan	ZnO NP	5%	5%	5%	5 wt% ZnO	^75^
Sunscreen cream	Lignin	15–45%	20–60%	25–80%	10 wt% Lignin	^76^
PPC	TiO_2_/lignin	7–70%	13–70%	13–70%	5 wt% lignin-TiO_2_	^77^
PECA–PPC	Caffeic acid	0	0	17%	2% caffeic acid	^78^
CMC	CuO NP	0	1%	0.08–1%	8 wt% CuO	^51^
CMC	CuO@ZnO core/shell NPs	0	0	0	4 wt% CuO@ZnO core/shell NPs	Present work

*PVA* Polyvinyl alcohol, *PMMA* Polymethyl methacrylate.

##### Sunscreen performance of different CMC/ 4wt.% CuO@ZnO hybrid nanocomposite samples

**Figure 16:**
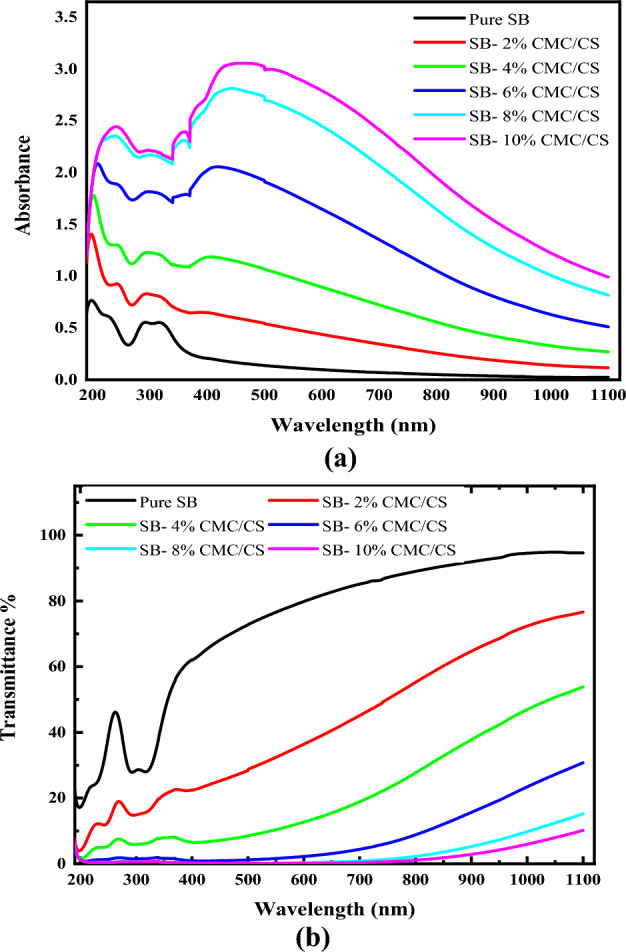
Variation of: (**a**) absorbance and (**b**) transmittance % as a function of wavelength for pure sunblock and sunblock mixed with different concentration of CMC/CuO@ZnO hybrid nanocomposite.

Sunblock and sunscreens are chemicals that block or absorb UV radiation and exhibit a range of immunosuppressive effects when exposed to sunlight. There are several UV-filtering agents available from both synthetic and natural sources. UV filters must be safe because they have the potential to cause significant human local and systemic exposure^34^. Synthetic UV filters have been shown to have potential toxicity in humans and to interfere only in specific pathways of the multistage process of carcinogenesis. Despite the abundance of UV-protecting materials, there is still a significant need for a natural substance that can replace these synthetic ones in a safe, non-toxic, and cost-effective manner and to satisfy Egypt's vision 2030 towards sustainable development^79,80^. Different CMC/4wt.% CuO@ZnO based sunscreens were prepared by mixing different concentration of the CMC nanocomposite solution with the commercial pure sunscreen cream. Figure 16a and b presents the variation of absorbance and transmittance with wavelength for pure Mash sunblock cream and sunblock mixed with different concentrations of CMC/4wt% CuO@ZnO hybrid nanocomposite solution. Figure 16a shows that the absorbance of the sunblock cream increased with increasing the concentration of CMC/4 wt% CuO@ZnO hybrid nanocomposite solution. The intensity of the characteristic absorption peaks of CMC and CuO@ZnO core/shell NPs increased with increasing CMC/4 wt% CuO@ZnO hybrid nanocomposite concentration in the sunblock. The letters confirm the miscibility of the CMC/4 wt% CuO@ZnO Mash sunblock solution. Figure 16b confirms the suitability of CMC/4 wt% CuO@ZnO hybrid nanocomposite as a blocking material in personal care products as the transmittance of pure sunblock cream decreased sharply until it reached to 1% (see Table 9).

**Table 9:** UV blocking percentage of CMC/CuO@ZnO sunblock cream.

Sunblock loaded with CMC/4 wt% core/shell	UV blocking %		
	UVC	UVB	UVA
0	67–83%	67–72%	38–72%
2	84–96%	84%	78–84%
4	94–98%	94%	94%
6	98–99%	98%	98–99%
8	99%	99%	99%
10	99%	99%	99%

Additionally, the figure shows that the UV blocking of the pure Mash sunblock was enhanced and extended to the visible and near IR regions with the addition of 6, 8, and 10 wt% of CMC/4 wt% CuO@ZnO hybrid nanocomposite.

Sun protection factor (SPF) is the ratio of UV energy required to produce a minimal erythemal dose (MED) in protected skin versus unprotected skin. Screening the absorbance of the product between 290 and 320 nm at 5 nm intervals is a simple, quick, and reliable in vitro method of calculating the SPF. SPF can be calculated using the Mansur equation and the following formula^81^.

18 $$SPF=CFx{\sum }_{290}^{320}EE \left(\lambda \right)xI \left(\lambda \right)x Abs \left(\lambda \right)$$

where CF = correction factor (10), EE(λ) = erythmogenic effect of radiation with wavelength (λ), and Abs (λ) = spectrophotometric absorbance values at certain wavelength. The values of EE (λ) x I(λ) are fixed. The SPF of the pure cream was found to be 5.46, whereas the SPF of the creams containing various amounts of CMC/CuO@ZnO polymer nanocomposite (PNC) increased to 8.21, 12.24, 18.12, 21.67, and 22.10 for 2, 4, 6, 8, and 10% of CMC/CuO@ZnO (Table 10). As the CMC/CuO@ZnO content increased to 10% results in a better sunscreen performance, as the SPF value increased to 22.10, an increase of 404% over the pure cream. This phenomenon was primarily related to the size of CuO@ZnO core/shell nanoparticles, small size improved sunscreen performance. The significant sunscreen performance of CMC/4 wt% CuO@ZnO was possibly related to the conjugated system in CMC/4 wt%CuO@ZnO. Furthermore, the Zn=O, and Cu=O groups, can contribute significantly to the conjugated system in CMC (C=O). Furthermore, the presence of π–π* transitions between the aromatic rings in CMC and the sunscreen cream would benefit the sunscreen performance. However, compared to some earlier reports, this study's SPF values were lower this is due to the very small concentration of the sunscreen was used. Furthermore, the chemical sunscreen used in earlier studies has excellent sunscreen properties by nature. According to the annual sunscreen report published by World Health Organization, they recommended sunblock creams with SPF ranges from 15 to 50^82^. Accordingly, the Mash/CMC/4wt.% CuO@ZnO hybrid nanocomposite can be used as a sunblock.

**Table 10:** The SPF values and the absorbance of commercial sunscreen and CMC/4wt.% CuO@ZnO based sunscreens in the UVB region.

Wavelength (nm)	EExI	Absorbance					
		Pure SB	SB-2 wt% PNC	SB-4 wt% PNC	SB-6 wt% PNC	SB-8 wt% PNC	SB-10 wt% PNC
290	0.015	0.553	0.825	1.222	1.806	2.163	2.208
295	0.0817	0.553	0.831	1.230	1.815	2.168	2.216
300	0.2874	0.546	0.827	1.228	1.817	2.170	2.216
305	0.3278	0.543	0.820	1.224	1.814	2.168	2.209
310	0.1864	0.547	0.815	1.219	1.809	2.165	2.206
315	0.0837	0.552	0.809	1.213	1.803	2.160	2.197
320	0.0180	0.550	0.798	1.201	1.792	2.144	2.184
SPF		5.465	8.207	12.235	18.122	21.670	22.096

## Conclusion

Based on the obtained results it can be concluded that CuO@ZnO core/shell nanoparticles were successfully synthesized using the Co-precipitation method meanwhile, nanocomposite films based on CMC were successfully synthesized using the casting method. XRD analysis revealed an increase in the amorphous nature of the nanocomposite films with the addition of metal oxide NPs, confirming the formation of well-structured nanocomposite films. HR-TEM analysis confirmed the formation of core/shell nanoparticles, while FTIR analysis indicated the presence of hydrogen bonding between the hydroxyl group of CMC and the oxygen atom of the prepared nano metal oxides, signifying the successful formation of nanocomposites. UV–Vis results demonstrated a redshift in the absorption edge as the NP content increased, indicating a decrease in the optical bandgap energy. The type of transition in CMC was identified as an indirect allowed transition.

The concentration of core/shell NPs in the CMC nanocomposites affected various material characteristics. An increase in the values of refractive index was observed, a decrease in oscillation energy and an increase in dispersion energy with increasing core/shell NPs concentration. The lattice dielectric constant was determined to have higher values than the infinite high-frequency dielectric constant, indicating the presence of free carrier contribution. The calculated Urbach energy, which measures disorder within the material, increased as the concentration of metal oxide NPs increased.

The tunable optical characteristics of CMC/CuO@ZnO core/shell NPs nanocomposites make them promise for various optoelectronic applications. The nanocomposite films containing different concentrations of CuO@ZnO core/shell NPs showed a high blocking capacity for UV radiation, making them suitable candidates for UV-shielding applications. Optical experiments revealed that pure CMC films exhibited varying transmittance levels within the UVC, UVB, and UVA ranges. Increasing the concentration of core/shell NPs led to a reduction in transmittance within the UVC, UVB, and UVA ranges. The addition of 4 wt% CuO@ZnO core/shell NPs to the CMC matrix significantly decreased transmittance in both the UV and visible regions, with approximately 100% of UV light being absorbed. The CMC/CuO@ZnO core/shell hybrid nanocomposite exhibited a significantly higher sun protection factor (SPF) compared to pure sunblock cream, meeting the requirements set by the World Health Organization for preferred sunblock.

## Data Availability

The data will be available upon request. Contact Medhat A. Ibrahim, Email: medahmed6@yahoo.com.
